# Targeting Inflammatory Pathways in Chronic Low Back Pain: Opportunities for Novel Therapeutics

**DOI:** 10.3390/ph18111612

**Published:** 2025-10-24

**Authors:** Panagiota Anyfanti, Paschalis Evangelidis, Konstantinos Tragiannidis, Christina Antza, Dimitrios Poulis, Theodoros Dimitroulas, Vasilios Kotsis

**Affiliations:** 1 Third Department of Internal Medicine, Papageorgiou Hospital, Aristotle University of Thessaloniki, 54124 Thessaloniki, Greece; panyfan@hotmail.com (P.A.); konstantinos.tragiannidis@gmail.com (K.T.); kris-antza@hotmail.com (C.A.); dppoulis@gmail.com (D.P.); 2 Second Propedeutic Department of Internal Medicine, Hippoκration Hospital, Aristotle University of Thessaloniki, 54124 Thessaloniki, Greece; pascevan@auth.gr; 3 Fourth Department of Internal Medicine, Hippokration Hospital, Aristotle University of Thessaloniki, 54124 Thessaloniki, Greece; dimitroul@hotmail.com

**Keywords:** anti-NGF, anti-TNF, CRP, inflammation, LBP, proinflammatory cytokines

## Abstract

Low back pain (LBP) is a highly prevalent musculoskeletal problem and a leading cause of disability worldwide. From a pathophysiological perspective, the contribution of inflammation to LBP is being increasingly recognized. In this literature review, we aim to provide an overview of the role of inflammation as a mediator of LBP while summarizing clinical studies investigating the potential role of anti-inflammatory treatments in the management of LBP. Although often controversial, the available evidence suggests an important role of inflammation in the pathogenesis of LBP, which can be further translated into novel therapeutic targets. Both anti-tumor necrosis factor (anti-TNF) and anti-nerve growth factor (anti-NGF) agents hold the potential of blocking inflammation and pain pathways in patients with chronic LBP. TNF inhibitors have been tested mostly in small trials with mixed results, and their long-term efficacy remains to be proven. Anti-NGF agents have demonstrated stronger and consistent efficacy in randomized controlled trials, but safety concerns compromise their widespread use. The potential role of other anti-inflammatory molecules is currently under investigation. Presently, the routine use of TNF or NGF inhibitors is not supported in radiculopathy or chronic LBP. However, novel anti-inflammatory therapies introduced in the rheumatology field appear to be promising for specific subsets of patients suffering from chronic, refractory LBP, with a complementary role as therapeutic tools, after the unsuccessful outcome of the conservative approach.

## 1. Introduction

Low back pain (LBP) is a highly prevalent musculoskeletal problem and a major public health problem across all ages and socioeconomic strata worldwide. In 2020, LBP affected 619 million people globally, with an increasing prevalence among older decades that compromises the prospect of healthy ageing. It has been further projected that LBP will reach 843 million prevalent cases by 2050, which corresponds to a 36.4% increase from 2020 [1]. This continuously increasing trend is consistent with the growth in the global population and increasing life expectancy. The magnitude of the problem is reflected in years lived with disability due to LBP. According to current estimates, LBP is recognized as a leading cause of disability in both developed and developing countries, which exerts major limitations on daily activities, mobility, and work ability and severely affects emotional status and health-related quality of life [1,2,3]. LBP is associated with remarkably high rates of hospitalization and costs, which rise continuously with increasing rates of unindicated imaging, high rates of surgery, and subsequent revision surgery that unfortunately fail to eliminate or efficiently address the problem [4]. Importantly, there is evidence from the UK Biobank analysis suggesting that chronic LBP exacerbates cardiovascular morbidity and mortality independent of established cardiovascular risk factors, socioeconomic factors, comorbidities, and medication [5]. Underlying mechanisms involve sympathetic nervous system activation as a stress response to chronic pain, increased prevalence of shared cardiovascular risk factors such as hypertension, obesity, aging, and sedentary lifestyle, and chronic subclinical inflammation that is detrimental to the vasculature [6,7,8,9]. The effect of commonly prescribed drugs for chronic LBP needs to be further taken into account, with the most pronounced example of non-steroidal anti-inflammatory drugs (NSAIDs) that are known to increase blood pressure and contribute to cardiovascular morbidity and mortality [10,11].

LBP is not a disease but a symptom resulting from several, often heterogeneous, clinical conditions or diseases, defined by the location of pain (typically between the lower rib margins and the buttock creases) and commonly accompanied by expansion of the pain to one or both legs, with or without neurological symptoms in the lower limbs [12]. LBP is caused by irritation of nerve roots and is roughly classified as specific and non-specific. Specific causes of LBP include a broad spectrum of traumatic, degenerative, oncologic, infectious, inflammatory, metabolic, postural, and congenital etiologies resulting from a definitive pathology (i.e., vertebral fracture, malignancy, spinal infection, axial spondylarthritis, ankylosing spondylitis, cauda equina syndrome, etc.) [13,14]. Non-specific LBP cannot be attributed to a known specific pathology and encompasses a wide range of degenerative and idiopathic conditions, but the most common causes of LBP are disc herniation and spinal stenosis.

LBP may be further categorized based on the pattern of pain (axial, radicular, or referred), duration, characteristics (nociceptive or neuropathic), and episodic nature (flare-up or recurrent) of the symptoms [15]. Specifically, based on the duration of LBP, it can be classified into three distinct types: acute LBP (pain lasting <6 weeks), subacute LBP (6–12 weeks), and chronic LBP (>12 weeks, despite adequate care) [16]. LBP might present as episodic (recurrent), with distinct pain episodes and symptom-free time intervals, or as persistent (continuous), with enduring discomfort whose intensity varies. Typically, episodic LBP is self-limiting and mechanical, while persistent patterns are usually considered indicative of degenerative, inflammatory, or central sensitization underlying causes [17,18]. Moreover, LBP can be classified by its clinical presentation [19,20]. Mechanical LBP worsens with movement, whereas it eases with rest. On the contrary, inflammatory LBP is characterized by morning stiffness and improvement during exercise. Neurogenic LBP, mainly due to spinal stenosis, appears while standing or walking, subsiding on sitting or flexion. Pain from facet or discogenic causes usually radiates to the gluteal or hip regions. Axial pain is localized to the lumbar region without radiation, arising from spinal structures, such as intervertebral discs or facet joints [19]. In contrast, radicular pain results from nerve root irritation or compression, often accompanied by sensory or motor deficits. Radicular symptoms commonly manifest as sharp, burning, or electric-like pain radiating along a dermatome, combined with tingling or even muscle weakness, differentiating them from the aching, localized pain of nociceptive origin. Additionally, lumbosciatic pain refers to LBP with radiation to the sciatic pathway—from the lumbar region to the gluteal area and the posterior thigh or leg, mainly due to compression from disc herniation or radiculopathy [21]. As such, LBP can be a response to degeneration, inflammation, instability, or a combination of all, often severely limiting clinicians’ ability to diagnose the exact pathologic source of these symptoms and offer an appropriate or definite cure [22]. Until today, as described above, the diagnosis of LBP remains clinical and is based on a detailed history, clinical examination, assessment of red-flag symptoms, and imaging techniques (for selected cases). No validated biomarkers are currently used in the routine definitive diagnosis of LBP.

Although mechanical nerve root compression has been traditionally considered as the major cause of disc-induced radicular pain, recent experimental and clinical observations have suggested an important role of inflammatory mediators in the pathogenesis of LBP, which irritate and damage nerve roots [23]. Local production of proinflammatory cytokines [tumor-necrosis factor alpha (TNF-α), interleukin-1 (IL-1)] is upregulated in animal models of degenerated and herniated discs, specifically within the nucleus pulposus, Schwann cells, and epidural space [24]. Endoneural injection of TNF-a in rodents promotes vascular and histological nerve modifications resembling those relevant to extrusion of nucleus pulposus [25], while experimental TNF-a blockade prevents disc herniation, nerve sensitization, and ingrowth [26]. These observations provide the background for the exploitation of anti-inflammatory treatments towards non-conventional management of LBP in clinical practice. Although inflammatory pathways have been a main target of treatment for patients with systemic autoimmune, chronic inflammatory diseases, clinical studies support a potential role for selected cases of LBP as well. Therefore, in the present review article, we aim to provide an overview of the role of inflammation as an important mediator of LBP while summarizing clinical studies supporting a potential role of anti-inflammatory treatments in the management of chronic LBP. Special emphasis will be placed on the most mature anti-inflammatory treatments for management of chronic LBP, i.e., anti-TNF-α inhibitors and anti-nerve growth factors (NGFs).

## 2. Anatomy and Degeneration Characteristics of Disc Herniation as a Cause of Chronic LBP

**Figure 1:**
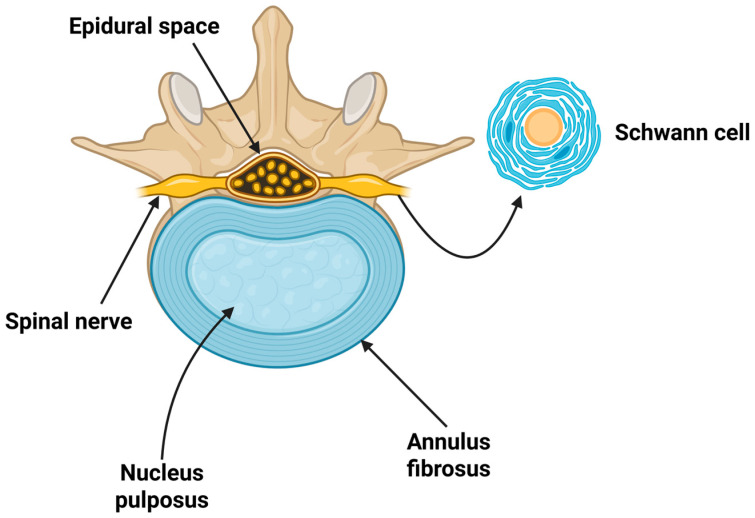
An overview of the intervertebral disc. In this figure, the intervertebral disc (nucleus pulposus and annulus fibrosus), spinal nerves, and epidural space are presented, highlighting anatomical sites associated with radicular pain. Created in BioRender. Evangelidis, P. (2025), https://biorender.com/npwbi4l, accessed on 11 October 2025.

Intervertebral disc degeneration is a major cause of LBP. The intervertebral disc has a crucial role in the connection of vertebral bodies and is composed of three fundamental parts: nucleus pulposus, annulus fibrosus, and cartilaginous endplate (Figure 1) [27]. Nucleus pulposus, located in the middle, is rich in water-rich proteoglycans and collagen, mainly type I, which absorb the applied pressure, while producing the components of the extracellular matrix [28]. The cartilaginous endplate surrounds the two remaining parts, limiting the diffusion of nutrients within the interior of the disc. Importantly, intervertebral discs lack nerves and a blood supply, and, thus, are “inflammation-protected”.

Older age is considered a significant risk factor for disc degeneration due to various contributing factors, including mechanical and oxidative stress [29]. This can be attributed to a decrease in nucleus pulposus cells, characterized by an impaired ability to synthesize collagen type II in combination with a lower concentration of proteoglycans, resulting in a disproportion in the extracellular matrix [30]. Thus, the mechanical properties of the disc are diminished. Another important factor implicated is the fibrosis and calcification of the cartilaginous endplate, which impede the diffusion of essential nutrients. Moreover, in the degenerated discs, atypical development of blood vessels occurs, and, thus, they become susceptible to inflammatory damage, along with abnormal growth of nerve fibers associated with sensitivity to pain [31]. As mentioned above, apoptosis of nucleus pulposus cells has a major role in disc degeneration development. Oxidative stress and increased mechanical strain can activate signaling pathways, such as mitochondrial dysfunction, through a Bax/Bcl-2 imbalance and the release of cytochrome C, ultimately leading to caspase-dependent cell death [32]. These processes result not only in the impairment of extracellular matrix production but also in the release of pro-inflammatory molecules, contributing to the local activation of inflammatory responses and promoting degeneration [33].

**Figure 2:**
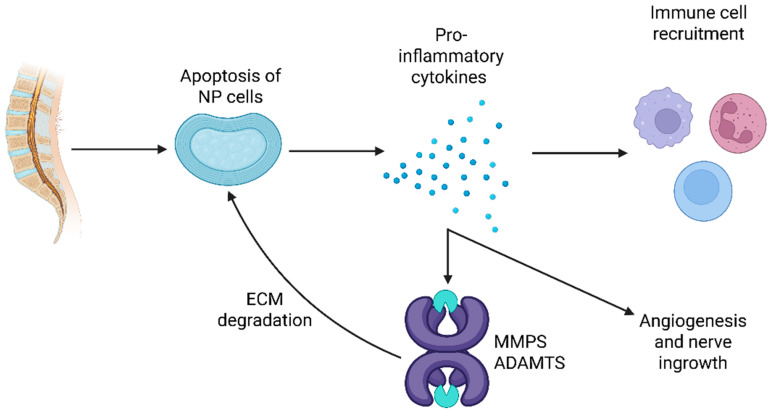
Pathogenic loops of inflammation, degeneration, and pain in CLBP. Apoptosis of NP cells initiates the release of pro-inflammatory cytokines (activation of NF-κB and MAPK pathways), while attracting immune cells to the intervertebral disc. These cytokines also activate MMPs, leading to ECM degradation and loss of disc integrity. The resulting tissue breakdown promotes angiogenesis (via VEGF signaling) and nerve ingrowth (via NGF signaling) into normally “aneural” regions of the disc. Created in BioRender. Evangelidis, P. (2025) https://BioRender.com/sor8781, accessed on 11 October 2025. ADAMTS: a disintegrin and metalloproteinase with thrombospondin motifs, CLBP: chronic low back pain, ECM: extracellular matrix, MMPs: matrix metalloproteinases, MAPK: mitogen-activated protein kinase, NGF: nerve growth factor, NF-κB: nuclear factor kappa B, NP: nucleus pulposus, VEGF: vascular endothelial growth factor.

As shown in Figure 2, injured or apoptotic nucleus pulposus cells release pro-inflammatory cytokines and danger signal patterns, which can activate nuclear factor kappa (NF-κB) and mitogen-activated protein kinase (MAPK) pathways, leading to recruitment of inflammatory cells and degradation of the extracellular matrix [34]. This immune dysregulation is further enhanced by angiogenesis and nerve ingrowth mediated by vascular endothelial growth (VEGF) and nerve growth factor (NGF), respectively, resulting in a vicious cycle of inflammation, structural damage, and pain sensitization.

## 3. Systemic Inflammation in Patients with Chronic LBP

Several inflammatory circulatory biomarkers have been investigated in patients with chronic LBP. The role of C-reactive protein (CRP) and high-sensitivity CRP (hs-CRP) has been explored in several studies, exhibiting contradictory findings [35].

### 3.1. CRP and hs-CRP

Gebhardt et al., in their prospective longitudinal study, included 41 patients with chronic LBP and 31 with acute lumbosciatic pain, followed up for 6 months, aiming to investigate systemic inflammation using hs-CRP [36]. Chronic LBP was defined as pain persisting for ≥3 months without radicular symptoms, as assessed with visual pain analogue scales and functional questionnaires. In the group of LBP patients, hs-CRP levels were low and stable for the whole study period, with no statistically significant changes over time. Additionally, hs-CRP, in this patient population, was not associated with intensity of pain, functional capacity, or clinical outcomes. However, in another cross-sectional study, hs-CRP was increased in comparison to pain-free controls, while associated with pain severity in LBP subjects [37]. We have to underline that hs-CRP is characterized by higher sensitivity in comparison to CRP in the detection of low-grade inflammation [38]. More studies, with a large number of patients, are essential to elucidate the role of hs-CRP in LBP. For this aim, collaboration with primary care units can be helpful.

In a cross-sectional analysis of 50,666 participants from rural areas in Norway aged between 20 and 96 years, Ho and colleagues aimed to identify the potential correlation among sleep disturbances, chronic LBP, and systemic inflammation, as depicted by CRP levels [39]. Chronic LBP was defined as LBP or stiffness with a duration of more than 3 months. Interestingly, an association was reported between CRP levels and the presence of chronic LBP (odds ratio: 1.01, *p* = 0.013). Moreover, in a large biobank analysis in the United Kingdom, CRP levels were compared between three groups: chronic LBP, acute LBP (ALBP), and pain-free controls. Participants with chronic LBP had significantly elevated CRP levels (LBP: 2.18 ± 3.73 mg/L, ALBP: 2.04 ± 3.45, controls: 1.95 ± 3.42 mg/L, *p* ≤ 0.001 in each comparison). Thus, these findings from epidemiological studies demonstrate CRP as an emerging and potential marker of systemic inflammation in chronic LBP [40].

### 3.2. Inflammatory Cytokines

Various inflammatory molecules, such as cytokines, chemokines, and endothelial injury molecules, have been studied in the field of chronic LBP. In the case–control study of Kraychete et al., it was shown that in patients with chronic LBP due to disc herniation, levels of TNF-α (*p* = 0.01) and interleukin-6 (IL-6) (*p* = 0.06) were higher in comparison to healthy controls. Nevertheless, no statistically significant differences were identified in interleukin-1 (IL-1) and soluble TNF receptor (sTNF-R) serum levels among the two groups [41]. Li et al. analyzed peripheral blood samples from a patient cohort with chronic LBP and age-matched healthy controls to explore potential markers of systemic inflammation in LBP [42]. Increased expression of IL-6 and decreased levels of interleukin-10 (IL-10), an anti-inflammatory cytokine, were reported in the LBP cohort in comparison to healthy controls. Interestingly, CD16+ monocytes, a cell population that induces pro-inflammatory responses, were elevated, while the capacity for β-endorphin secretion in M2-type macrophages (typically anti-inflammatory) was lower in the LBP group. Moreover, expression of repulsive guidance molecule b (RGMb), known as Dragon, was found to be reduced in M1 macrophages.

Pedersen and colleagues conducted a 12-month prospective study of people with lumbar radicular pain, attributed to disc herniation, aiming to identify the role of IL-6 and IL-8 in pain chronicity [43]. Interestingly, IL-8 appeared especially as a biomarker for pain persistence and chronicity. Specifically, IL-8 remained elevated over the follow-up period and was associated with pain severity. In the research work of Luchting et al., expression levels of the P2X7 receptor (P2X7R) and interleukin-1β (IL-1β) in the peripheral blood were compared between patients with chronic LBP, neuropathic pain (NeP), and healthy controls [44]. The rationale of these markers’ investigation was based on the fact that activation of P2X7R promotes the release of IL-1β via the inflammasome, contributing, among others, to pain sensitization [45]. However, no significant differences in IL-1β and P2X7R levels were found between patients with chronic LBP and healthy controls. Yet, we have to recognize that the sample size of the study was small, and, thus, the role of these markers should be investigated also in future research in larger cohorts.

Teodorczyk-Injeyan and colleagues investigated the inflammatory profile of patients with LBP, comparing the findings between acute and chronic LBP cases with healthy controls [46]. In this study, it was found that both acute and chronic LBP patients were characterized by significantly increased levels of pro-inflammatory cytokines, such as TNF-α, IL-1β, and IL-6, along with elevated ratios of these cytokines to the anti-inflammatory cytokine IL-10, in comparison to healthy controls. Additionally, interferon-γ expression was lower in both patient groups compared to controls, while it was positively associated with pain severity scores. Importantly, in the chronic LBP cohort, the production of TNFα, IL-1 receptor antagonist, and sTNF-R was significantly higher than in the other two cohorts. Moreover, in chronic LBP patients, TNFα and IL-1β levels were positively correlated with pain intensity, as measured by the visual analogue scale (VAS). In another work of this group, the association between chronic LBP versus ALBP and healthy controls and elevated inflammatory biomarkers, including cysteine-cysteine motif (CC) chemokines and soluble E-selectin (sE-selectin), was investigated [47]. Compared with pain-free controls, both ALBP and LBP subjects exhibited significantly increased in vitro production of CC chemokine ligands 2 and 4. Interestingly, plasma levels of sE-selectin were increased considerably in chronic LBP patients (*p* = 0.003), but not in ALBP. However, the increased levels of sE-selectin, a marker of endothelial injury, in patients with chronic LBP compared to the rest, might be attributed to the potentially high cardiovascular disease burden that these patients experience. Dadkhah and colleagues in their case–control study aimed to elucidate the relationship between serum levels of inflammatory mediators, among other molecules, with chronic LBP and pain severity [37]. Specifically, chronic LBP patients exhibited elevated levels of inflammatory mediators, such as IL-1β and TNF-α. Furthermore, the severity of chronic LBP, as assessed by the McGill and Oswestry questionnaires, was correlated significantly with IL-1β and TNF-α.

Xu et al. conducted a cross-sectional study to identify the possible relationship between inflammatory markers, 25-hydroxyvitamin D [25(OH)D] levels, and pain severity in patients with non-specific LBP [48]. In their work, 60 patients with ALBP, 78 patients with chronic LBP, and 60 healthy controls were included. It was shown that serum concentrations of 25(OH)D were significantly lower in the chronic LBP cohort compared to the control group (LBP group: 18.25 ± 8.05 ng/mL, controls: 25.70 ± 10.04 ng/mL, *p* < 0.001). Moreover, IL-6 levels were elevated in subjects with chronic LBP compared to pain-free controls (LBP: 4.68 ± 2.56 pg/mL, controls: 2.98 ± 1.45 pg/mL, *p* < 0.001), while IL-6 levels were found to be associated with increased pain intensity and disability rates. In multivariate analysis, it was shown that IL-6 levels, independently of vitamin D, were correlated with chronic LBP. In the case–control study of Slouma et al., 46 subjects with chronic LBP were included, along with 60 matched for age and gender healthy controls [49]. In their analysis, IL-8 was significantly increased in the LBP cohort compared to controls. Moreover, IL-8 was associated with the VAS radicular pain score.

Gjefsen and colleagues aimed to identify serum inflammatory biomarkers in patients with chronic LBP who had type 1 and 2 modic changes (MCs) on magnetic resonance imaging (MRI), distinguishing them from healthy subjects [50]. MCs are defined as vertebral endplate and bone marrow lesions, representing a subcategory of chronic LBP. Among 40 serum cytokines and chemokines measured, macrophage migration inhibitory factor (MIF) was characterized by the strongest ability for separation of patients and controls in receiver operating characteristic (ROC) analysis [area under the curve (AUC) = 0.79].

Nevertheless, in some studies, the hypothesis regarding the increased levels of proinflammatory cytokines in patients with chronic LBP has not been validated. For instance, in the case–control study of Heffner and colleagues, 25 adults with chronic LBP were compared to 25 pain-free controls, aiming to investigate whether sleep disturbance is associated with circulating IL-6 levels, as a surrogate marker of systemic inflammation [51]. Specifically, IL-6 levels did not differ significantly between the chronic LBP patients and the pain-free controls. However, in the LBP cohort, poor sleep quality and greater pain severity were correlated with elevated IL-6 levels. Similarly, in the study of Capossela et al., IL-6 levels were not statistically different between chronic LBP subjects and healthy controls [52]. Likewise, levels of various other pro-inflammatory molecules, including IL-1β and TNF-α, were similar among the two cohorts. However, certain anti-inflammatory molecules, such as interleukin-4 (IL-4) and granulocyte colony-stimulating factor, were diminished in LBP patients. These findings might be explained by the fact that long-term administration of anti-inflammatory agents might suppress cytokine expression, or even that chronic pain could downregulate pro-inflammatory signaling. A significant limitation of this study was the relatively small and heterogeneous populations included. In Table 1, we provide a summary of the studies investigating inflammatory biomarkers in patients with chronic LBP.

In summary, there is emerging evidence indicating that chronic LBP is associated with an imbalance among pro- and anti-inflammatory molecules. Pro-inflammatory cytokines, including TNF-α, IL-1β, and IL-6, have a major role in the sensitization of nociceptors of pain, promoting disc degeneration and leading to systemic inflammation, while IL-8 further enhances this process by the recruitment of neutrophils. Circulating chemokines, such as CCL2 (MCP-1) and CCL4, contribute to the recruitment and migration of inflammatory cells and pain signaling. This is reflected by the elevated proportion of CD16+ pro-inflammatory monocytes, as has been shown in chronic LBP subjects in comparison to healthy controls. Systemic inflammatory markers such as CRP and hs-CRP, a more sensitive variant, indicate the state of low-grade inflammatory burden, while increased levels of sTNF-R and sE-selectin are considered as markers of TNF pathway activation and endothelial injury, respectively [53]. On the contrary, levels of anti-inflammatory cytokines, such as IL-4 and IL-10, are often low, highlighting the impaired regulation of inflammatory processes. Together, this profile suggests a shift toward a pro-inflammatory state in chronic LBP, contributing to pain chronicity and tissue damage. In Table 2, these findings are summarized.

## 4. The Role of Modern Therapies in the Management of Chronic LBP

In most cases, a conservative approach is first preferred for the management of CLBP, given that it is the safest therapy, without any possible complications or excessive cost, intending to relieve the symptoms and control the pain [54]. Conservative treatment options include both non-pharmacological, such as spinal manipulation, massage, heat, dry needling, acupuncture, psychological intervention, exercise, and physical therapy, and pharmacological therapies (mostly NSAIDs) [31,55]. However, conservative treatment is not effective for some patients, especially in cases of severe or prolonged mechanical pain [56]. For patients with refractory LBP and functional disabilities, surgery may be considered, although not preferred [31]. For these patients, modern anti-inflammatory therapies may provide a novel route for LBP management. The most mature and widely studied biological therapies for LBP target inflammatory pathways, involving anti-TNF-α and anti-NGF agents [23].

### 4.1. Anti-TNF-α Inhibition

Anti-TNF-α biologic agents act by targeting TNF-α, a key cytokine, as described above, in the development of inflammation and sensitization of pain through activation of the NF-κB signaling pathway and pro-inflammatory cytokine release [57]. In chronic LBP, TNF-α release from degenerated discs and immune cells promotes inflammation and nerve root irritation, while its blockade can potentially reverse these processes [58]. Etanercept, infliximab, adalimumab, golimumab, and certolizumab pegol are all approved anti-TNF-α inhibitors for several clinical entities, whereas their usage has been associated with improvements in mechanical pain, while many other agents are under experimental use [59,60].

Etanercept is an sTNFR construct that can be efficient in patients with radiculopathy, especially when used epidurally [61]. Infliximab is a chimeric monoclonal antibody that is administered intravenously and has debatable results against chronic LBP. Some studies do not support the use of infliximab for LBP, since in comparison with placebo, it reported no different results [62,63]. Adalimumab and golimumab are two fully human monoclonal antibodies [59]. Adalimumab is injected subcutaneously and can show improvement against chronic LBP [64]. Golimumab is also injected subcutaneously and is approved as a therapy against various rheumatic diseases, both in adults and pediatrics. It can be used either as a monotherapy or as a part of a combination therapy with methotrexate against inflammatory arthritis [65]. Last, certolizumab pegol is an antigen-binding fragment of a recombinant human monoclonal antibody that neutralizes TNF-α [66]. It is also recommended against inflammatory autoimmune diseases and is especially effective for psoriatic arthritis [67].

Anti-TNFα therapy could be considered effective for cases of patients with confirmed inflammatory LBP, following unsuccessful conservative management and NSAIDs administration. This therapy results in rapid pain relief and improves the quality of life for the patients some weeks after its initiation, while also preventing irreversible structural damage in the axial skeleton [68]. Although patients with mostly non-specific LBP do not effectively respond to the anti-TNF-α therapy, this treatment constitutes a promising approach against LBP, given its positive results in functional improvement and pain management [58]. However, most trials were small, heterogeneous, and short-term, providing mixed results. Randomized controlled trials (RCTs) evaluating the use of TNF-α inhibitors for the management of LBP are summarized in Table 3. Although some early improvements were observed in pain and function, sustained benefit over placebo was not consistently shown. However, outcomes were likely influenced by route of administration (systemic vs. epidural vs. perispinal) and patient selection. Hence, routine use of anti-TNF agents for nonspecific LBP outside research settings is not supported by current evidence. More well-designed studies, with multicenter collaboration, are essential in this clinical field.

### 4.2. Anti-NGFs

Anti-NGFs provide another modern option for the management of LBP. NGF is a neurotrophic factor that was discovered around 70 years ago, for its action on survival and differentiation that takes place in the peripheral neurons. Elevated levels of NGF have been found in degenerative disks and facet joints in patients with LBP [77,78]. NGF mediates the overexpression of proinflammatory neuron-derived molecules such as substance P, serotonin, and calcitonin gene-related peptide [79]. Its therapeutic properties can have a clinical impact on multiple diseases, such as Alzheimer’s and brain traumas, eye diseases, but also in osteoarthritis and rheumatic diseases [78,80,81,82]. Tanezumab, fulranumab, and fasinumab are humanized monoclonal antibodies inhibiting NGF [83,84,85]. Tanezumab is the most effective and widely used among these antibodies. Its efficacy was confirmed by several studies in the past, and it was evaluated for chronic LBP in as many as thousands of patients [86,87]. Tahir et al., after analyzing data from 4514 patients, characterized tanezumab administration as effective with a proposed dose from 5 to 10 mg [86]. Another study by Lian et al. included 3414 patients and focused on the dosages, while also mentioning the efficacy of the drug [88]. It also highlighted that intravenous and subcutaneous injections had a positive impact on chronic LBP. Some other studies recommend even higher dosages of 20 mg, given the higher responding rates in the clinical trials [89]. On the other hand, according to other scientific references, there is evidence that tanezumab should not be recommended, due to low to moderate pain relief and some safety concerns [90].

Fulranumab is another human recombinant monoclonal antibody with therapeutic properties for multiple conditions. It is presented as an option for LBP, but it is still under investigational use, and its effectiveness is still debatable [91]. A phase II trial that tested dosages between 1 and 10 mg every 4 weeks showed long-term sustained improvements in all efficacy parameters following fulranumab treatment [92]. On the other hand, another phase II study, which included 385 patients, highlights that this therapy was well-tolerated, but no differences in outcomes were observed as compared to the placebo [93]. However, fulranumab is a drug that has reported important improvements in the therapy of severe pain conditions, such as osteoarthritis [91]. Fasinumab, an investigational NGF inhibitor, can also be effective for the management of multiple diseases. Focusing on the chronic LBP, results from studies are characterizing it as effective with significant functional and pain improvement. The clinical study by Dakin et al. reported the data of 563 patients with chronic LBP and also mentioned the need for higher doses to have satisfactory results [94]. According to the results of another study with randomized fasinumab doses between 0.1 mg/kg and 0.3 mg/kg, there was no difference between the drug and placebo therapy [94]. However, fasinumab, as well as tanezumab and fulranumab, are showing a great therapeutic potential, and all these agents are considered very promising for the management of chronic LBP and other diseases. A special note is needed for tanezumab, which is thought to be the most effective monoclonal antibody. Its effectiveness is really hopeful for many patients who have a downgrade in their quality of life, suffering from severe LBP [95].

Table 4 summarizes RCTs evaluating the use of anti-NGFs for the management of LBP. Multiple RCTs have demonstrated efficacy, with significant pain reduction and improved function in chronic LBP patients. Despite the satisfactory levels of efficacy, symptom relief, and the general improvement in the quality of life, safety concerns have been raised. The development of rapidly progressive osteoarthritis and risk for joint replacement limits their clinical use [96]. Further concerns have been raised regarding the combination of anti-NGF therapy with the administration of NSAIDs [84]. Consequently, clinical development was halted by the Food and Drug Administration in 2021, despite strong analgesic signals. These adverse effects are common and can be irreversible, but correct and careful dosing can minimize the risk. Further clinical studies and trials should take place in order to better estimate the balance between efficacy and safety of this therapy and finally draw firm conclusions regarding the profile of patients with chronic LBP that would be ideal candidates for anti-NGF administration.

### 4.3. Other Molecules

For patients who do not effectively respond to the previous standard therapies for LBP, there are some other molecules that could be used as potential treatment options. Janus kinase (JAK) inhibitors are a relatively new class of small-molecule, oral biologic disease-modifying antirheumatic drugs that block the Janus kinase/signal transducer and activator of transcription (JAK-STAT) signaling pathway [101]. JAK inhibitors have been widely used over the last years for the treatment of inflammatory arthropathies, including rheumatoid arthritis and axial spondylarthritis [102,103]. JAK inhibitors may have a role in the amelioration of LBP of various etiologies. Preclinical models of non-inflammatory LBP have demonstrated that JAK/STAT signaling is upregulated and functionally contributes to the nociplastic pain (mechanical, degenerative) independent of classic inflammation [104,105,106]. This is further supported by the rapid pain relief achieved by JAK inhibitors in patients with spondylarthritis, including, in terms of pain, outcomes beyond just inflammation [106,107].

In comparison to anti-TNF and anti-NGF biologics, JAK inhibitors are characterized by a broader molecular action mechanism, having an impact on several cytokine pathways via the blockade of the JAK-STAT signaling pathway, and, thus, potentially being efficient for both inflammatory and central sensitization pain [108]. It has to be underlined that JAK inhibitors are administered orally with a rapid onset. As mentioned above, while anti-TNF have been associated with positive treatment outcomes mainly in inflammatory LBP, whereas anti-NGF monoclonal antibodies are strong analgesics, JAK inhibitors might offer a balanced combination of these two effects. Nevertheless, more clinical and mechanistic data are crucial for these questions.

Furthermore, there are some modulators of inflammation, such as hydroxytyrosol, that reduce proinflammatory cytokines like TNF-α and also decrease the expression of NGF [109,110]. Moreover, there are some other drugs, reparixin (interleukin-8 receptor inhibitor) and cyclooxygenase (COX-2) inhibitors, that reduce the neuronal inflammation and have already been reported to have beneficial results against the chronic LBP [111]. Symptomatic slow-acting drugs administrated for generalized osteoarthritis may also partially improve LBP of degenerative origin, representing an additional complementary approach [112].

ECM might serve as an important therapeutic target. ECM-based hydrogels limit the nerve ingrowth into degenerating disks, while additionally delivering the regenerative factors that help restore disc function and reduce the pain [109]. However, clinical applicability has not been extensively studied, and further research is needed to lead to safe conclusions about it.

Lastly, neurotrophic factors include NGF and brain-derived neurotrophic factor (BDNF), which are upregulated during disc degeneration and promote nerve ingrowth and pain. The blockage of these molecules, by using antibodies against NGF or its receptors, reduces pain-related nerve activity in animal models [113,114]. This method is effective pre-clinically, but there are several concerns about the clinical outcomes.

## 5. Conclusions and Future Perspectives

For several decades, LBP has been ranked as a main cause of disability worldwide, and it is still considered a major public health problem, with a noticeable impact on the quality of life for many patients [1,2,3,4]. For patients with refractory or recurrent LBP in whom current medical and non-pharmaceutical interventions fail to provide pain relief, there is an emerging need for novel therapies. A thorough understanding of the underlying pathophysiological mechanisms may provide further insights into the modern therapeutic options. Experimental and clinical data point towards an important role of inflammation in the pathogenesis of LBP, which can be further translated into novel therapeutic targets. Both anti-TNF-α and anti-NGF agents hold the potential of blocking inflammation and pain pathways in LBP. TNF-α inhibitors have shown efficacy signals, although these are debatable and largely inconsistent. On the other hand, anti-NGF agents are more efficient, yet their clinical exploitation is compromised by serious adverse events. Nevertheless, several limitations should be recognized in these studies: small sample size, heterogeneous populations, different treatment endpoints, lack of mechanistic and molecular data, and short-term follow-up periods. While other molecules are currently under investigational use, future studies should focus on identifying subgroups of patients with LBP that may benefit safely from advanced treatments, including NGF and TNF-α inhibition. Moreover, future research in this field should examine the potential effectiveness of NSAIDs, novel anti-inflammatory biologics, and physical therapy combinations in the management of CLBP. Another interesting research idea would be the investigation of proteomic/genomic factors, such as single-nucleotide polymorphisms, implicated in the treatment response of patients who receive biologic agents for chronic LBP. Similar studies in the field of autoimmune diseases can be inspirational for reaching this aim [115].

## Figures and Tables

**Table 1:** A summary of studies investigating inflammatory biomarkers in patients with low back pain. Several markers of inflammation have been investigated in patients with CLBP.

Reference	Study Design	Number of Participants	Age (years), Mean (SD)	Female Sex, *n* (%)	Duration of Pain	CLBP Evaluation	Biomarkers
Gebhardt, et al., 2006 [36]	Prospective longitudinal comparative study	-72 cases (31: with ALBP, 41: CLBP) -1572 controls (from the VERA study)	-ALBP group: 44.8 (20–64) -CLBP: 42.2 (27–57)	-ALBP: 16 (51.6) -CLBP: 27 (65.8)	-ALBP: <8 weeks -CLBP: >3 months	-VAS -Hannover questionnaire	hs-CRP
Kraychete et al., 2010 [41]	Case–control study	-23 cases with CLBP -10 controls	-CLBP: 42.8 (7) -Controls: 39.5 (4.5)	-CLBP: 11 (47.8) -Controls: 4 (40)	≥3 months	NRS	IL-1, IL-6, TNF-α, sTNF-R
Heffner et al., 2011 [51]	Case–control study	-25 cases with CLBP -25 controls	30.8 (11.4)	-Total cohort: 30 (60)	≥6 months	MPQ-SF	IL-6
Pedersen et al., 2015 [43]	Prospective longitudinal	127 patients with LBP due to disc herniation	40 (10)	65 (51.2)	>1 month	VAS	IL-6, IL-8
Li et al., 2016 [42]	Case–control study	-35 cases with CLBP -35 controls	Range: 45–70	NS	NS	NS	IL-6, IL-10, monocyte markers (CD14, CD16), β-endorphin
Luchting et al., 2016 [44]	Case–control	-19 cases with CLBP -19 cases with NeP -19 controls	-CLBP: 40 (11) -NeP: 47 (13) -Controls: 58 (13)	-CLBP: 15 (79) -NeP: 13 (68) -Controls: 11 (58)	NS	PainDETECT-questionnaire	P2X7R, IL-1β
Capossela et al., 2018 [52]	Case–control	-23 cases with CLB -30 controls	52.5 (15.9)	-CLBP: 14 (61) -Controls: NS	NS	NS	IL-6, IL-1β, TNF-α, IL-2, IL-10, MCP1, CCL5, CXCL6, G-CSF
Teodorczyk-Injeyan et al., 2018 [47]	Non-randomized clinical trial	-19 cases with ALBP patients -23 cases with CLBP -21 controls	-ALBP: 35.4 (9.9) -CLBP: 31.6 (7.8) -Controls: 36.1 (11.4)	-ALBP: 7 (36.9) -CLBP: 10 (43.5) -Controls: 8 (38.1)	-ALBP: <4 weeks -CLBP: ≥12 weeks	-VAS -Oswestry disability index	CCL2, CCL3, CCL4, sE-selectin
Teodorczyk-Injeyan et al., 2019 [46]	Case–control	-22 cases with ALBP -25 cases with CLBP -24 controls	-ALBP: 32.8 (9.2) -CLBP: 36.5 (11.1) -Controls: 35.2 (10.4)	-ALBP: 9 (40.9) -CLBP: 11 (44) -Controls: 9 (37.5)	-ALBP: <4 weeks -CLBP: >12 weeks	-VAS -Oswestry disability index	TNFα, IL-1β, IL-6, IL-2, IL-10, IL-1 receptor antagonist, sTNF-R2
Ho et al., 2019 [39]	Cross-sectional study	6559 with CLBP	52.6	16,663 (54)	>3 months	Standardized Nordic questionnaire for musculoskeletal symptoms	CRP
Dadkhah et al., 2020 [37]	Case–control study	-148 cases with CLBP -150 controls	-CLBP: 49.2 (6.1) -Controls: 47.57 (5.8)	-CLBP: 87 (50.3) -Controls: 86 (49.7)	>12 weeks	-MPQ -Oswestry disability index	IL-1B, IL-6, hs-CRP, TNF-α
Xu et al., 2021 [48]	Case–control study	-60 cases with ALBP -78 cases with CLBP -60 controls	-ALBP: 64.46 (10.45) -CLBP: 63.17 (12.49)-Controls: 62.31 (11.06)	-ALBP: 43 (71.7) -CLBP: 51 (65.4) -Controls: 32 (53.3)	-ALBP: <12 weeks -CLBP: >12 weeks	-VAS -Modified Oswestry disability index	CRP, WBCs, neutrophils, TNF-α, IL-6, IL-1, 25(OH)D,
Gjefsen et al., 2021 [50]	Case–control study	-46 cases with CLBP and MC1 -37 cases with CLBP and MC2 -50 controls	-MC1: 42.1 (8.3) -MC2: 45.8 (9.0) -Control: NS	-MC1: 32 (69.6) -MC2: 19 (51.4)	>6 months	-NRS -Oswestry Disability Index	40 different cytokines
Slouma et al., 2023 [49]	Case–control study	-46 CLBP cases -60 controls	-CLBP: 43.17 (8.7) -Controls: NS	NS	>3 months	VAS	IL-6, IL-8, IL-17, IL-23, IL-22, TNF-α
Hodges et al., 2023 [40]	Case–control study	-Initial cohort: 59,208 (CLBP: 17,642, ALBP: 11,962, controls: 29,604) -Validation cohort: 2326 (CLBP: 669, ALBP: 494, Controls: 1163)	-Initial: 55.8 (8.1) -Validation: 60.7 (7.6)	Initial cohort: -CLBP: 8586 (48.7%) -ALBP: 5348 (44.7%) -Controls: 13,934 (47.1%) Validation cohort -CLBP: 303 (45.3%) -ALBP: 207 (41.9%) -Controls: 510 (43.	>3 months	Self-reported	CRP

ALBP: acute lower back pain, CCL: CC chemokine ligand, CLBP: chronic low back pain, CXCL: C-X-C motif chemokine ligand 10, G-CSF: granulocyte colony-stimulating factor, hs-CRP: high-sensitivity C-reactive protein, IL-1: interleukin-1, IL-10: interleukin-10, IL-17: interleukin-17, IL-2: interleukin-2, IL-22: interleukin-22, IL-23: interleukin-23, IL-6: interleukin-6, IL-8: interleukin-8, CP1: monocyte chemoattractant protein 1, MCs: modic changes, MPQ-SF: McGill pain questionnaire short form, NeP: neuropathic pain, NRS: numeric rating scale, NS: not stated, P2X purinoceptor 7: P2X purinoceptor 7, sE-selectin: soluble E-selectin, sTNF-R: soluble tumor necrosis factor receptor, TNF-α: tumor necrosis factor-α, VAS: visual analogue scale, WBC: white blood cells.

**Table 2:** Circulating inflammatory markers in chronic LBP.

Marker	Biological Role	Findings	Reference
hs-CRP	Acute-phase protein Sensitive marker of low-grade systemic inflammation	Increased Association with pain severity	[36]
CRP	Acute-phase protein	Increased Association with sleep disturbance	[39]
TNF-α	Pro-inflammatory cytokine Induces of production of IL-1β, IL-6, chemokines, and adhesion molecules	Increased Association with pain severity	[37]
sTNF-R	Cleavage of the extracellular domains of TNF receptors circulating in plasma	Increased	[41]
IL-1β	Pro-inflammatory cytokine promoting prostaglandin synthesis and leukocyte recruitment	Increased Association with pain severity	[37]
IL-6	Pro-inflammatory cytokine Key stimulator of hepatic CRP production	Increased Association with pain severity, disability rate, and sleep disturbance	[37]
IL-8	Chemokine recruiting neutrophils	Increased Association with pain severity	[43]
MCP-1	CC chemokine recruiting monocytes and memory T cells Role in neuroinflammation	Increased	[42]
CCL4	CC chemokine attracting monocytes, NK cells, and T cells	Increased	[42]
CD16+ monocytes	Pro-inflammatory monocytes with enhanced capacity for producing pro-inflammatory cytokines	Increased	[42]
sE-selectin	Adhesion molecule Mediation of leukocyte adhesion and migration	Increased	[47]
IL-4	Anti-inflammatory cytokine	Decreased	[52]
IL-10	Anti-inflammatory cytokine	Decreased	[42]

CCL4: chemokine ligand 4, CD16+: cluster of differentiation 16, CRP: C-reactive protein, hs-CRP: high-sensitivity C-reactive protein, IL-1β: interleukin-1 beta, IL-4: interleukin-4, IL-6: interleukin-6, IL-8: interleukin-8, IL-10: interkeukin-10, interleukin-10, LBP: low back pain, MCP-1: monocyte chemoattractant protein-1, NK: natural killer, sE-selectin: soluble E-selectin, sTNF-R: soluble tumor necrosis factor receptor, TNF-α: tumor necrosis factor-alpha.

**Table 3:** RCTs investigating the use of anti-TNF therapy in patients with LBP.

Author/Year	Population	Study Design	Anti-TNF Agent	Main Outcomes	Key Findings
Cohen et al., 2012 [69]	84 adults with lumbosacral radiculopathy of less than 6 months’ duration	Multicenter, triple-arm RCT	Etanercept (epidural)	The primary outcome measure was leg pain 1 month after the second injection.	-Small differences favoring steroids compared with saline and etanercept were observed for back pain. -Etanercept fared worse for functional capacity than the other treatments (steroids and placebo)
Genevay et al., 2010 [70]	61 patients with acute (duration of <12 weeks) and severe (ODI score of >50) radicular leg pain and imaging-confirmed lumbar disc herniation	RCT, placebo-controlled	Etanercept (subcutaneous, repeated)	The primary outcome was the VAS score for leg pain.	-Small but significant improvement in leg pain. -Patients receiving adalimumab demonstrated significantly better outcomes in terms of back pain and a reduced rate of surgery.
Okoro et al., 2010 [71]	15 patients with acute unilateral radicular leg pain secondary to a herniated nucleus pulposus, confirmed on magnetic resonance imaging scan	Triple blind, placebo-controlled RCT	Etanercept (subcutaneous injection in the perispinal area)	Primary outcome measures were VAS pain, ODI score, modified somatic perception, and modified Zung depression index	No benefit to the use of etanercept over placebo, but small numbers of trial participants limited statistical analysis.
Cohen et al., 2009 [72]	24 patients with subacute lumbosacral radiculopathy	RCT, placebo-controlled	Etanercept (epidural)	The primary outcome was a numerical rating scale leg pain score reflecting pain. Secondary outcome measures included ODI score, numerical rating scale back pain score, reduction in analgesic medications, and global perceived effect.	Significant improvements in leg and back pain for the etanercept-treated patients, but not for the placebo group, one month after treatment.
Cohen et al., 2007 [73]	36 patients with chronic lumbosacral radiculopathy or discogenic LBP	RCT, placebo-controlled	Etanercept intradiscally	The primary outcome was the VAS pain score. Secondary outcome measures included ODI score, reduction in analgesic medications, and global perceived effect.	-A single low dose of intradiscal etanercept was not effective for chronic radicular or discogenic LBP according to pain scores or disability scores, which did not differ between or within groups for any dose range or subgroup of patients.
Korhonen et al., 2005 [74]	40 patients with unilateral moderate to severe sciatic pain with an MRI-confirmed disc herniation	Randomized, double-blind, placebo-controlled	Infliximab IV (single dose)	The primary endpoint was a reduction in leg pain through 12 weeks.	-No differences vs. placebo in the primary endpoint. -No differences were observed in the secondary outcomes (reduction in back pain and ODI score, improvement of straight leg restriction, differences in the number of days on sick leave, and the number of discectomies).
Gjefsen et al., 2025 [62]	128 patients with moderate to severe chronic low-back pain and Modic type 1 changes	Randomized, triple-blind, placebo-controlled, multicenter trial	Infliximab (four IV infusions 5 mg/kg) over ~5 months	No significant difference in the primary outcome between infliximab and placebo at 5 months.	-Secondary outcomes reported no effect. -Adverse event rates were similar between groups.
Genevay et al., 2012 [75]	61 patients were enrolled (31 in the adalimumab group and 30 in the placebo group) with acute and severe sciatica	Multicenter, randomized, double-blind, placebo-controlled trial	Adalimumab (40 mg, 2 subcutaneous injections, within 2 weeks)	Primary short-term outcome: leg pain (measured by visual analogue scale, VAS 0–100 mm), daily for 10 days, then at 6 weeks and 6 months. Long-term (3-year) outcome: incidence of discectomy	-Over time, the adalimumab group had a more favorable course of leg pain vs. placebo. -Fewer surgical discectomies in the adalimumab group vs. placebo (6 vs. 13) in the short/medium-term follow-up.
Ohtori et al., 2012 [76]	80 patients with lumbar spinal stenosis (40 patients received etanercept, 40 patients received dexamethasone)	Prospective, randomized, controlled study	Etanercept (epidural administration onto the spinal nerves)	Epidural administration of etanercept was more effective than dexamethasone for leg pain, low back pain, and leg numbness. No adverse event was reported in either group.	Epidural administration of a TNF inhibitor onto the spinal nerve led to pain relief, without adverse events.

IV: intravenous, LBP: low back pain, kg: kilogram, MRI: magnetic resonance imaging, mg: milligram, ODI: Oswestry Disability Index, RCT: randomized controlled trial, TNF: tumor necrosis factor, VAS: visual analogue scale, VS: versus.

**Table 4:** Randomized controlled trials investigating the use of anti-NGF agents in patients with LBP.

Author/Year	Population	Study Design	Anti-NGF Agent	Main Outcomes	Key Findings
Sanga et al., 2016 [93]	183 patients with chronic LBP	Phase II, randomized, double-blind, placebo-controlled, dose-ranging study	Fulranumab (SC, every 4 weeks)	Primary endpoint: pain improvement at week 12	-Fulranumab was characterized as well-tolerated, but significant results compared with placebo were not reported. -Neurologic TEAEs were more frequent in fulranumab group (25%) vs. placebo (14%)
Katz et al., 2011 [95]	217 adult patients, with chronic LBP (72 received tanezumab, 73 received oral naproxen, 72 placebo)	Randomized, double-blind, multicenter, placebo-controlled trial.	Tanezumab, IV (200 µg/kg–single dose)	LBPI at week 6	-Tanezumab was more effective against pain and reported improvements in disability. -No severe TEAEs were mentioned, but 9/72 patients discontinued.
Kivitz et al., 2013 [97]	1347 patients with chronic LBP	Phase IIb randomized, placebo-controlled trial	Tanezumab IV (5, 10, or 20 mg every 8 weeks)	The primary endpoint was the mean change in daily average LBPI from baseline to week 16	-10 and 20 mg of tanezumab had similar efficacy and improved LBPI. -Arthralgia and paresthesia were the most frequent TEAEs, resulting in discontinuation.
Gimbel et al., 2014 [98]	849 patients with chronic LBP	Non-controlled, randomized, multicenter study	Tanezumab (IV injections of 10 or 20 mg)	The primary outcome was average pain	-A dose of 10 mg proved better tolerated than 20 mg. -No severe TEAEs were reported.
Markman et al., 2020 [99]	1825 participants were enrolled (406: placebo group, 407: tanezumab 5 mg, 407: tanezumab 10 mg, 605: tramadol prolonged-release 100–300 mg/day)	Randomized, double-blind, placebo- and active-controlled, phase 3 study	Tanezumab (5 or 10 mg SC every 8 weeks)	Reported efficacy with ≥50% LBPI reduction at week 16	-Tanezumab 5 mg did not meet the primary endpoint. -TEAEs were more frequent at the 10 mg dose.
Dakin et al., 2021 [94]	563 patients with moderate to severe chronic LBP	Phase II/III, double-blind, placebo-controlled study	Fasinumab (SC 6 mg or 9 mg or IV 9 mg)	The primary outcome was the change from baseline to week 16 in average daily LBPI	-Significant placebo-adjusted LBP reductions at week 16 for fasinumab 9 mg SC and 9 mg IV. -6 mg dose was not significantly better. -Joint TEAEs were more common in fasinumab vs. placebo.
Tiseo et al., 2014 [100]	159 adults with moderate-to-severe unilateral sciatica pain	Randomized, double-blind, placebo-controlled, parallel-group proof-of-concept trial	Fasinumab (0.1 or 0.3 mg/kg SC)	AUC of average leg pain scores (measured by numerical rating scale) from baseline to Week 4	-No notable difference in average leg pain reduction compared with placebo. -More TEAEs occurred with fasinumab, especially at the higher dose.

AUC: area under the curve, IV: intravenous, LBP: low back pain, kg: kilograms, LBPI: low back pain intensity, mg: milligrams, μg: micrograms, NGF: nerve growth factor, SC: subcutaneously, TEAE: treatment-emergent adverse event.

## Data Availability

No new data were created or analyzed in this study. Data sharing is not applicable to this article.
